# Efficacy of Magnesium Supplementation in Functional Constipation: A Systematic Review

**DOI:** 10.7759/cureus.116715

**Published:** 2026-09-22

**Authors:** Hasan Kadhem, Ward Alasfoor, Mohamed Saeed Ali, Talal Sarhan, Vijayalakshmi S Bhojaraja, Jeevan K Shetty

**Affiliations:** 1 Medical School, Royal College of Surgeons in Ireland - Bahrain, Busaiteen, BHR; 2 Medical School, Faculty of Medicine, Mansoura University, Mansoura, EGY; 3 Anatomy, Royal College of Surgeons in Ireland - Bahrain, Busaiteen, BHR; 4 Biochemistry, Royal College of Surgeons in Ireland - Bahrain, Busaiteen, BHR

**Keywords:** functional bowel disorders, magnesium oxide, quality of life, stool consistency, stool frequency

## Abstract

Magnesium supplementation has been proposed as a therapeutic option for functional constipation due to its osmotic properties, role in smooth muscle contraction, and influence on aquaporin-3 expression; however, the evidence has not been systematically synthesized. This review assessed whether magnesium supplementation, in any formulation, improves stool frequency, stool consistency, or quality of life in patients of any age with functional constipation, compared with placebo, standard care, or an active comparator. A systematic search of PubMed, Scopus, Web of Science, EMBASE, and CINAHL was conducted over a 10-year period to December 2025. Study quality was assessed utilizing the Mixed Methods Appraisal Tool. Due to substantial heterogeneity in the studies, a narrative synthesis was performed. This review was reported in accordance with the Preferred Reporting Items for Systematic Reviews and Meta-Analyses statement. The certainty of the evidence was not formally graded. Seven studies (n = 690) met the inclusion criteria, addressing functional constipation across infant, pediatric, and adult populations. Five were randomized controlled trials, and two were nonrandomized studies. Four of seven studies showed statistically significant between-group improvements in stool frequency. Six studies reported stool consistency numerically, with statistically significant between-group differences in four; the remaining studies showed either within-group improvement or no significant differences between groups. Constipation-specific quality-of-life testing was assessed using a validated instrument in only two studies, both of which showed significant between-group improvement. Magnesium oxide was the most commonly used formulation in adults. No serious adverse events were reported in any trial, although adverse event reporting was inconsistent across studies. The findings suggest that magnesium supplementation, primarily magnesium oxide, may improve outcomes in functional constipation, but are not sufficient to support a treatment recommendation. The findings relate to magnesium oxide and cannot be assumed to apply to other forms of magnesium supplementation. The evidence base is limited by small sample sizes, heterogeneous study designs, and confounded interventions. Double-blind, placebo-controlled trials with standardized outcomes are needed to further explore this domain.

## Introduction and background

Magnesium is the fourth most abundant element and the second most abundant cation in the human body [1]. It plays a prominent role in biochemical reactions in the body, serving as a cofactor for over 600 enzymes [1,2]. Magnesium absorption primarily occurs in the distal small intestine, with passive diffusion through the paracellular pathway being the main mechanism, although active transport has been observed in the descending colon [2,3].

Given its direct relevance to gastrointestinal tract function and disease, magnesium is vital for smooth muscle function, the predominant muscle type in the gastrointestinal tract [4,5]. The Mg-ATP complex is required for numerous cellular processes and, in smooth muscle, is essential for regulating muscle contraction [2,4]. Furthermore, magnesium plays a role in regulating aquaporin-3, as increasing cellular Mg^2+^ increases aquaporin-3 expression [6]. This is directly relevant to the gastrointestinal tract, as decreased aquaporin-3 has been implicated in inflammatory diseases such as irritable bowel syndrome (IBS) and inflammatory bowel disease (IBD) [7,8].

Moreover, magnesium levels affect calcium homeostasis, a key second messenger in the immune system [5]. In the human gastrointestinal tract, magnesium has been shown to play a potential role in functional bowel disorders (FBDs), which are gastrointestinal disorders with symptoms attributed to the middle or lower gastrointestinal tract [9,10]. These include IBS, functional bloating, functional constipation, functional diarrhea, and unspecified FBDs. Magnesium has been proposed as a treatment option for functional constipation, as magnesium salts are poorly absorbed osmotic agents that influence intestinal water handling [11].

Functional constipation is among the most prevalent FBDs globally, with pooled prevalence estimates of approximately 10-15% depending on the diagnostic criteria used [12]. It is defined by the Rome IV criteria as recurrent difficult, infrequent, or incomplete defecation in the absence of an organic cause [10]. Osmotic laxatives are recommended as the first line, but response and adherence are often limited. Magnesium salts are widely used without a systematic appraisal of the evidence for them. This review, therefore, asks whether, in patients of any age with functional constipation, magnesium supplementation in any formulation or dose, compared with placebo, standard care, or an active comparator, improves stool frequency, stool consistency, or constipation-related quality of life.

## Review

Methodology

Protocol and Registration

This systematic review was reported in accordance with the Preferred Reporting Items for Systematic Reviews and Meta-Analyses (PRISMA) 2020 statement [13]. The review protocol was developed as part of the institutional research process at Royal College of Surgeons in Ireland - Bahrain and was approved by the Head of School in December 2025. A copy of the protocol is held by the institution and can be obtained from the authors on reasonable request. No changes were made to the protocol during the review. The review was not registered with PROSPERO because it did not meet the eligibility criteria at the time, and no other registry was used. The lack of prospective registration is acknowledged as a limitation, as it reduces transparency and the ability to assess potential selective outcome reporting.

Search Strategy

A systematic literature search was conducted across the following five major databases: PubMed, Scopus, Web of Science, EMBASE, and CINAHL. The search combined controlled vocabulary and free-text terms for magnesium with terms for FBDs and constipation. The full search strategy used for each database and the dates on which the searches were run are provided in the Appendices. The search was structured in the Population, Intervention, Comparison, Outcome (PICO) format, combining a population block, an intervention block naming magnesium and each commonly used magnesium salt, and a broad outcome block. Trial registries, conference proceedings, and other grey literature were not searched, and reference list screening and forward citation tracking were not undertaken.

The search was limited to the 10-year period preceding the search date (December 16, 2015 to December 16, 2025) and to human studies, with reviews and case reports excluded. This window was chosen to capture the contemporary evidence base and coincides with the introduction of the Rome IV criteria in 2016, including studies spanning the transition from Rome III to Rome IV. In PubMed, this limit was applied using the built-in 10-year publication date filter. In Scopus, Web of Science, EMBASE, and CINAHL, where only calendar-year limits were available, the search was restricted to 2015 to 2025, and records published before December 16, 2015 were excluded manually during screening.

Eligibility Criteria

Studies were eligible if they enrolled participants of any age with FBDs; evaluated magnesium supplementation in any formulation or dose; included a placebo, standard care (including standard infant formula), or an active comparator in routine clinical use; and reported at least one of stool frequency, stool consistency, or quality of life. Records were excluded if they were reviews, case reports, editorials, or conference abstracts, or if they did not evaluate magnesium supplementation, did not enroll a population with an FBD, lacked an eligible comparator, or reported none of the prespecified outcomes. Functional constipation was defined according to the Rome IV criteria [9] as difficult, infrequent, or incomplete defecation in the absence of an organic cause and not meeting criteria for IBS. Adult studies enrolling patients with chronic or chronic idiopathic constipation were considered to represent functional constipation where an organic cause was not identified. Constipation secondary to medications or to metabolic, neurological, or structural disease was not eligible. IBS-C, distinguished from functional constipation by predominant abdominal pain, was eligible as an FBD, but no studies of magnesium in IBS-C were identified.

Study Selection and Data Extraction

Titles and abstracts were screened independently by two reviewers, and the full texts of potentially eligible records were assessed independently by the same two reviewers. Covidence flagged all disagreements, which were resolved by discussion between the two reviewers. Data were independently extracted by two reviewers from each included study for the first author, year of publication, country, study design, population characteristics, magnesium formulation and dose, comparator, treatment duration, and outcomes related to stool frequency, stool consistency, quality of life, and adverse events. For each outcome domain, we sought all compatible results reported across all measures and time points, applying no restrictions by instrument or analysis. The searches were last run in December 2025. Covidence systematic review software (Veritas Health Innovation, Melbourne, Australia) was used for deduplication and screening management, and no automated eligibility classification tools were applied. All data supporting this review are derived from the published studies cited. No template data collection form or analytic code was generated. Study authors were not contacted for missing information. Full-text records excluded at the eligibility stage, with reasons, are listed in the Appendices.

Quality Appraisal

Study quality was assessed using the Mixed Methods Appraisal Tool (MMAT) 2018 version [14]. Each study was evaluated against two screening questions before proceeding to design-specific criteria: Category 2 for randomized controlled trials (RCTs) and Category 3 for nonrandomized quantitative studies. In accordance with MMAT guidance, overall numeric quality scores were not computed; individual criterion ratings are presented descriptively. The counts of criteria met are a descriptive tally of those ratings and are not a weighted quality score. Two reviewers independently performed the appraisals, and disagreements were resolved through discussion. Criterion-level judgments informed the weight given to each study in the synthesis, with greater weight given to double-blind trials reporting between-group comparisons. They were not used to exclude studies.

Data Synthesis

Due to substantial heterogeneity across the included studies in population age, magnesium formulation, dosage, comparator type, outcome measures, and study design, a meta-analysis was deemed inappropriate. Instead, a narrative synthesis was performed, with data organized by primary outcome and, where applicable, stratified by population subgroup. No common effect measure was calculated across studies; the effect estimates and p-values reported by each individual study are presented as published. No data conversions or imputations were performed. Sensitivity analyses were considered but were not feasible, given the small number of studies and the heterogeneity of populations, formulations, and outcome measures. The risk of bias due to missing results from reporting bias was not formally assessed. The certainty of the evidence was not formally graded, and no structured framework such as the Grading of Recommendations, Assessment, Development and Evaluations (GRADE) was applied.

Results

Search Results and Study Characteristics 

The systematic search yielded 522 records: Scopus (n = 302), PubMed (n = 75), Embase (n = 70), Web of Science (n = 55), and CINAHL (n = 20). After removal of 144 duplicate records, 378 records were screened by title and abstract, of which 362 were excluded. In total, 16 full-text articles were sought and retrieved, and all 16 were assessed for eligibility. Overall, nine were excluded for wrong intervention (n = 4), wrong study design (n = 2), wrong outcomes (n = 1), wrong patient population (n = 1), and wrong comparator (n = 1). Finally, seven studies met the inclusion criteria. The study selection process is summarized in the PRISMA diagram (Figure 1), and a completed PRISMA checklist is included in the Appendices.

**Figure 1:**
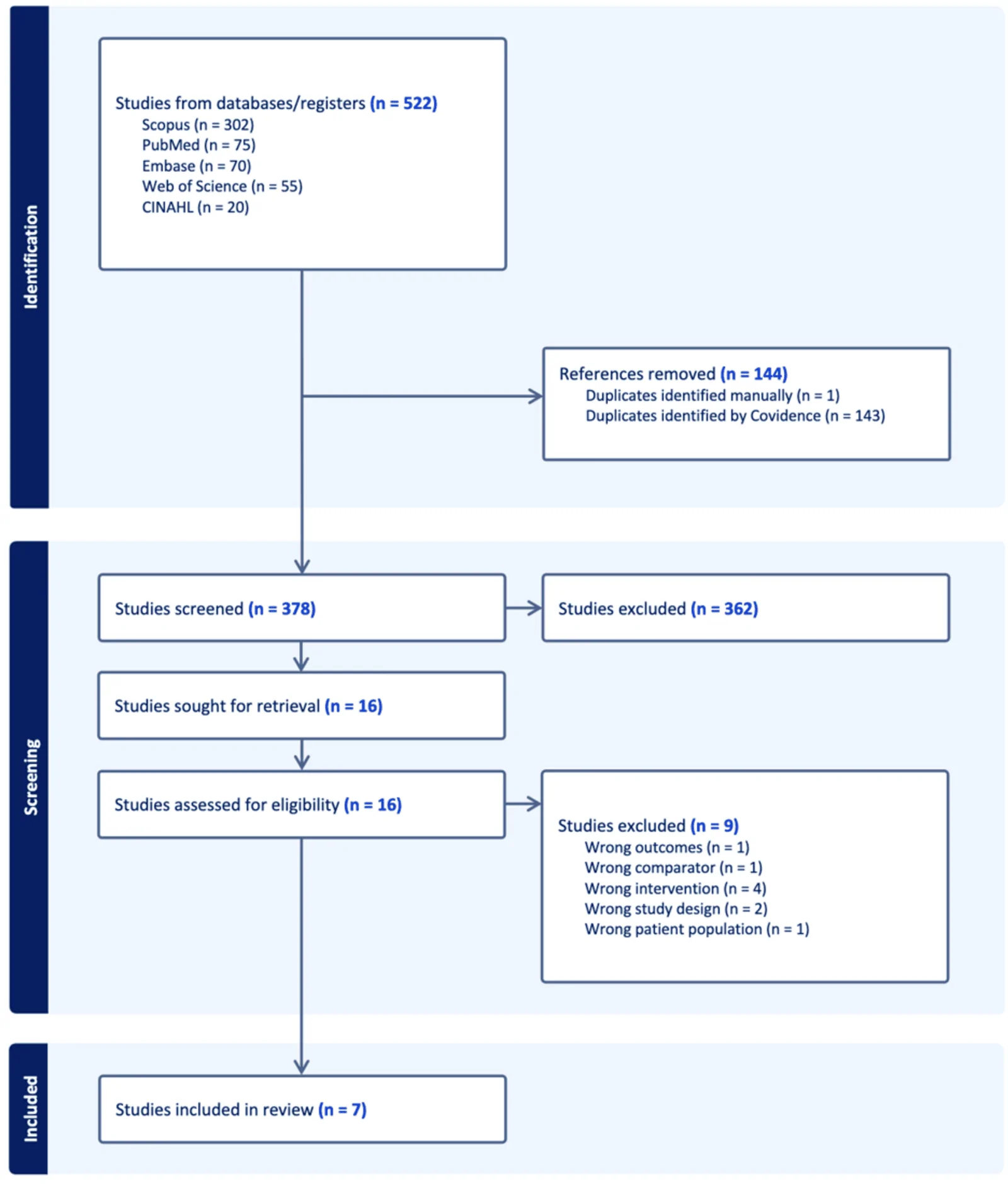
Preferred Reporting Items for Systematic Reviews and Meta-Analyses (PRISMA) 2020 flow diagram of study identification, screening, eligibility, and inclusion.

The seven studies included 690 patients, comprising five RCTs [15-19] and two nonrandomized studies. Three RCTs were double-blind [15,17,19], while the remaining two RCTs [16,18] and both nonrandomized studies [20,21] were open-label. The characteristics of all included studies are summarized in Table 1. Magnesium oxide was the most widely used formulation, appearing in four studies, while two studies used magnesium-enriched infant formulas and one used magnesium hydroxide.

**Table 1:** Characteristics of the included studies. Doses are reported as magnesium compounds rather than elemental magnesium unless otherwise specified. Elemental magnesium constitutes approximately 60% of the compound weight of magnesium oxide. Elemental magnesium was explicitly reported in only two of the included studies [17,19]; doses are therefore not directly comparable across formulations. DB = double-blind; F = female; Mg = magnesium; Mg(OH)₂ = magnesium hydroxide; MgO = magnesium oxide; mo = months; OL = open-label; PEG = polyethene glycol; RCT = randomized controlled trial; UAE = United Arab Emirates; wk = weeks; y = year

Author (year)	Country	Design	Population	Intervention	Comparator	Duration	Diagnostic criteria
Mori et al. (2019) [15]	Japan	DB RCT	n = 34, adults, mean age 40.6 y, 100% F	MgO 1,500 mg/day	Placebo	28 days	Rome IV
Benninga et al. (2019) [16]	UAE, Kuwait, Saudi Arabia, Oman, Lebanon	OL RCT	n = 285, infants, mean age ~5 mo	Mg-rich formula (8.5 mg/100 mL)	Standard formula	30 days	Modified Rome IV
Kubota et al. (2020) [17]	Japan	DB RCT	n = 60, children, mean age ~33–40 mo	MgO 30 mg/kg/day (elemental Mg ~18 mg/kg/day) ± *Limosilactobacillus reuteri*	*Limosilactobacillus reuteri* alone	4 weeks	Rome IV
Worona-Dibner et al. (2023) [18]	Mexico	OL RCT	n = 83, pediatric, 6 mo–18 y	Mg(OH)₂ 2 mL/kg/day	PEG 3350	12 months	Rome III
Morishita et al. (2021) [19]	Japan	DB RCT	n = 90, adults, mean age 42 y, 93.3% F	MgO 1,500 mg/day (elemental Mg ~900 mg/day)	Placebo; Senna	28 days	Rome IV
Kessoku et al. (2025) [20]	Japan	Prospective observational	n = 73, adults, mean age ~60 y, ~57% F	MgO mean 1,413 mg/day (compound weight; elemental content not reported)	Elobixibat	27 days	Rome IV
Xinias et al. (2018) [21]	Greece	OL non-randomized	n = 65, infants, mean age ~6–7 wk	Mg-enriched synbiotic formula (8 mg/100 mL)	Standard formula	1 month	Author-defined operational criteria

Quality Assessment

All seven studies passed both MMAT screening questions [14]. Among the five RCTs appraised under Category 2, methodological quality was variable: Kubota et al. [17] met all five criteria, Benninga et al. [16] and Morishita et al. [19] each met four, Mori et al. [15] met three, and Worona-Dibner et al. [18] met two. Key concerns included unclear randomization methods [15,18], baseline group imbalances [15], open-label designs precluding assessor blinding [16,18], and uncertain adherence due to protocol-permitted dose reductions [19]. Among the two nonrandomized studies (Category 3), both Kessoku et al. [20] and Xinias et al. [21] met three of five criteria, with shared limitations in representativeness and accounting for confounders. Individual criterion ratings are presented in Table 2.

**Table 2:** MMAT quality assessment ratings. Screening criteria applied to all studies: S1 = clear research question; S2 = collected data address the research question. ^a^Category 2 (randomized controlled trials): C1 = appropriate randomization; C2 = baseline comparability; C3 = complete outcome data; C4 = assessor blinding; C5 = participant adherence. ^b^Category 3 (non-randomized studies): C1 = representativeness; C2 = appropriate measurements; C3 = complete outcome data; C4 = confounders accounted for; C5 = intervention administered as intended. CT = can’t tell; MMAT = Mixed Methods Appraisal Tool; N = no; Y = yes

Study (year)	S1	S2	C1	C2	C3	C4	C5	Criteria met
Mori et al. (2019)^a^ [15]	Y	Y	CT	N	Y	Y	Y	3/5
Benninga et al. (2019)^a^ [16]	Y	Y	Y	Y	Y	N	Y	4/5
Kubota et al. (2020)^a^ [17]	Y	Y	Y	Y	Y	Y	Y	5/5
Worona-Dibner et al. (2023)^a^ [18]	Y	Y	CT	CT	Y	N	Y	2/5
Morishita et al. (2021)^a^ [19]	Y	Y	Y	Y	Y	Y	CT	4/5
Kessoku et al. (2025)^b^ [20]	Y	Y	CT	Y	CT	Y	Y	3/5
Xinias et al. (2018)^b^ [21]	Y	Y	CT	Y	Y	N	Y	3/5

Stool Frequency

All seven studies reported stool frequency outcomes (Table 3). Among adult populations that used magnesium oxide, magnesium supplementation was consistently associated with increased stool frequency compared with placebo. Mori et al. [15] demonstrated a clinically meaningful difference of approximately three additional spontaneous bowel movements (SBMs) per week, with a mean change of 6.07 ± 2.26 SBMs per week in the magnesium oxide group compared to 2.86 ± 2.42 SBMs per week in the placebo group (p = 0.002), a statistically significant between-group difference. Morishita et al. [19] reported an increase from four SBMs a week to nine with magnesium oxide, in comparison to 4.6 to approximately five with placebo (p < 0.001) for the between-group comparison. Complete SBMs per week increased from approximately 1.7 to approximately seven, versus from approximately 1.4 to approximately three. Kessoku et al. [20], which compared magnesium oxide to elobixibat, found no significant difference in SBMs (p = 0.172), with complete spontaneous bowel movements (CSBMs) favoring elobixibat (p = 0.013), both between-group comparisons.

**Table 3:** Summary of primary outcomes. Values are reported as published in the primary studies. Arrows (→) denote baseline to end-of-study values, and Δ denotes change from baseline. Treatment groups in Kubota et al. [17]: Gr A = *Limosilactobacillus reuteri* alone; Gr B = *Limosilactobacillus reuteri* + MgO; Gr C = MgO alone. BSFS = Bristol Stool Form Scale; CSBMs = complete spontaneous bowel movements; ctrl = control; elo = elobixibat; JPAC-QOL = Japanese Patient Assessment of Constipation Quality of Life; MCS = mental component summary; Mg = magnesium; MgO = magnesium oxide; MgOH = magnesium hydroxide; NS = not significant; PCS = physical component summary; PEG = polyethylene glycol; QoL = quality of life; SBMs = spontaneous bowel movements; SF-8 = Short Form-8 Health Survey; wk = week

Study (year)	Stool frequency	Stool consistency	Quality of life
Mori et al. (2019) [15]	SBMs/wk change: MgO 6.07 ± 2.26 vs placebo 2.86 ± 2.42 (p = 0.002)	BSFS change: MgO 2.83 ± 1.15 vs placebo 1.15 ± 1.03 (p < 0.001)	JPAC-QOL change: MgO 22.29 ± 13.13 vs placebo 6.31 ± 8.15 (p = 0.003); SF-8 PCS (p = 0.836), MCS (p = 0.165) NS
Benninga et al. (2019) [16]	≥1 stool/day by Day 29: Mg 99.3% vs ctrl 93.4% (p < 0.001)	Normal consistency Day 29: Mg 95.74% vs ctrl 64.96% (p < 0.001)	Not assessed (satisfaction only)
Kubota et al. (2020) [17]	Within-group ΔSBMs/wk at wk 4: Gr A +1.58, Gr B +1.64, Gr C +1.54 (all p ≤ 0.004); no between-group comparison	BSFS Δ at wk 4: Gr A +0.62 (NS), Gr B +0.88 (p = 0.017), Gr C +1.61 (p < 0.001)	Not assessed
Worona-Dibner et al. (2023) [18]	Not reported numerically; success: MgOH 95.24% vs PEG 97.56% (p = 0.616)	Not reported numerically	Not assessed
Morishita et al. (2021) [19]	SBMs/wk: MgO 4.0→9 vs placebo 4.6→5 (p < 0.001); CSBMs/wk: MgO 1.7→7 vs placebo 1.4→3 (p < 0.001)	BSFS: MgO 2.8→4–5 vs placebo 3.4 (p < 0.001)	JPAC-QOL total: MgO vs placebo (p < 0.001)
Kessoku et al. (2025) [20]	SBMs/wk: MgO 1.8→1.9 vs elo 2.0→2.6 (p = 0.172 NS); CSBMs: MgO 0.7→0.7 vs elo 0.7→1.9 (p = 0.013)	BSFS: MgO 1.5→1.9 vs elo 1.6→1.7 (p = 0.722 NS)	Satisfaction: MgO 47% vs elo 70% (p = 0.047)
Xinias et al. (2018) [21]	Median weekly freq: Mg 2.0→3.0 vs ctrl 1.0→1.0 (p < 0.001)	Hard stools: Mg 100%→27.5% vs ctrl 92%→92% (p < 0.001)	Parent QoL (non-validated): all 7 domains improved (p ≤ 0.002)

In infants, both of whom received magnesium-enriched formula rather than a magnesium salt, Benninga et al. [16] showed that 99.3% of infants receiving magnesium-rich formula improved, achieving ≥1 stool/day by day 29, compared with 93.4% of controls (p < 0.001). Xinias et al. [21] reported that the median weekly frequency increased from 2.0 to 3.0 in the treatment group, while the control group remained unchanged at 1.0 (p < 0.001). In pediatric populations, Kubota et al. [17], using magnesium oxide, reported statistically significant within-group improvements in all three arms at week four (Δ +1.54 to +1.64 per week; all 95% confidence intervals (CIs) excluding zero); however, the absence of between-group comparisons limits the ability to attribute the findings solely to magnesium. Worona-Dibner et al. [18] reported equivalent treatment success rates when comparing magnesium hydroxide and polyethylene glycol (PEG) 3350 (95.24% vs. 97.56%, p = 0.616).

Stool Consistency

All seven studies assessed stool consistency; however, different instruments were used between the studies. Four studies [15,17,19,20] used the Bristol Stool Form Scale (BSFS), a validated instrument, while the remainder used non-validated categorical descriptions of stool hardness, so results are not directly comparable across studies. Using the BSFS [22], Mori et al. [15] showed a clinically relevant shift from hard stools (Bristol types 1-2) to normal form (types 3-4), with a mean change of 2.83 ± 1.15 points in the magnesium oxide group versus 1.15 ± 1.03 in the placebo group (p < 0.001), a statistically significant between-group difference. Morishita et al. [19] observed BSFS scores increasing from 2.8 at initial measurement to approximately 4-5 with magnesium oxide (p < 0.001). Kubota et al. [17] reported that the magnesium oxide-alone arm achieved the largest within-group BSFS improvement (+1.61, 95% CI = 0.93 to 2.28, p < 0.001), followed by magnesium oxide + *Limosilactobacillus reuteri* (+0.88, 95% CI = 0.17 to 1.59, p = 0.017), while *L. reuteri* alone did not reach significance (+0.62, 95% CI = −0.07 to 1.31, p = 0.079); however, no between-group comparison was reported, so the improvement cannot be attributed to magnesium. The numerically larger changes in the magnesium oxide-containing arms are at most hypothesis-generating. Kessoku et al. [20] found no significant BSFS difference between magnesium oxide and elobixibat (p = 0.722). Other studies used non-validated categorical scales. Benninga et al. [16] reported a decrease in constipated stools from 89.51% to 2.84% in the magnesium group versus 90.85% to 33.58% in controls by day 29 (p < 0.001), with 95.74% achieving normal consistency. Xinias et al. [21] showed hard stools decreased from 100% to 27.5% in the intervention group while controls remained at 92% (p < 0.001).

Quality of Life

Quality of life was assessed utilizing validated instruments in only two studies (Table 3). Both enrolled Japanese adults received magnesium oxide, so these findings cannot be generalized to all patients with functional constipation. Mori et al. [15] employed the Japanese Patient Assessment of Constipation Quality of Life (JPAC-QOL) assessment, which reported a total change in the mean score of 22.29 ± 13.13 in the magnesium oxide group versus 6.31 ± 8.15 in the placebo group (p = 0.003). However, in the SF-8 Health Survey, there was no significant difference in the physical or mental component summaries, suggesting that the quality-of-life improvements were constipation-specific rather than reflecting changes in global health. Morishita et al. [19] also reported significant JPAC-QOL improvement favoring magnesium oxide over placebo (p < 0.001). The other studies, utilizing non-validated instruments, reported similar outcomes, although these are not directly comparable, with Xinias et al. [21] finding significant improvements across all seven parent-reported quality-of-life domains (p ≤ 0.002), while Kessoku et al. [20] reported higher treatment satisfaction with elobixibat than magnesium oxide (70% vs. 47%, p = 0.047).

Adverse Events

Adverse event reporting was inconsistent across studies. Three studies [15-17] reported no adverse events, though the methods of assessment varied in rigor. Morishita et al. [19] reported abdominal pain and diarrhea as the most common adverse events, with no serious adverse events. Dose reductions were required in 53.3% of magnesium oxide participants, 50% of placebo participants, and 83.3% of senna participants, suggesting that reductions were not specific to magnesium oxide treatment. Worona-Dibner et al. [18] found flatulence significantly more likely in the PEG group (40/40 vs. 37/42, p = 0.02), with more bowel movement pain with magnesium hydroxide (5/37 vs. 0/40, p = 0.02), but no severe events in either arm. Overall, magnesium supplementation was generally well tolerated across all age groups between the studies, with no serious adverse events reported in any trial. These data do not establish safety. Adverse event determination was inconsistent, and no included study was designed to detect uncommon harm. Magnesium is cleared renally, and hypermagnesemia is a recognized risk in patients with impaired renal function with high or prolonged dosing [24]. None of the studies enrolled patients with significant renal impairment. Dose-related diarrhea and abdominal pain were the most frequently reported effects. The high rates of dose reductions by Morishita et al. [19] indicate that tolerability at 1,500 mg/day is not uniform. The infant studies used magnesium-enriched formula rather than a pharmacological magnesium salt and therefore do not provide information on therapeutic dosing in infants. Worona-Dibner et al. [18] monitored serum magnesium and renal function at baseline and at three, six, nine, and 12 months, and recorded no cases of hypermagnesemia or hypomagnesemia in either arm. No other included study reported biochemical monitoring.

Discussion

Summary of Evidence

This systematic review synthesised seven studies evaluating magnesium supplementation for functional constipation, including 690 participants across infant, pediatric, and adult populations. The overall direction of the evidence demonstrated favorable results for magnesium supplementation, with four of seven studies showing statistically significant between-group improvements in stool frequency. Of the six studies that reported stool consistency numerically, four showed statistically significant differences between groups; the remainder showed only within-group change or no significant difference. Worona-Dibner et al. [18] did not report numerical consistency, and both studies using validated quality-of-life metrics showed significant improvements in quality of life.

There was substantial heterogeneity in the evidence base. The included studies compared various clinical populations from infants of approximately six weeks [21] to adults averaging 60 years. Formulations differed in dosing, and comparators included placebo, PEG, senna, and elobixibat over various durations. Study quality further compounds this, with the two studies offering the most direct evidence of a magnesium-specific effect in adults meeting only three [15] and four [19] of the five MMAT criteria; the single study meeting all five reported no between-group comparison [17]. Confidence in the overall direction of effect of magnesium for functional constipation is limited but reasonable; however, confidence in any specific magnitude, formulation, or dose-response relationship is not. These constraints apply throughout the following interpretation, which relies on seven small trials without quantitative pooling.

Mechanism of Action

The observed clinical improvements are biologically plausible and align with magnesium’s physiological roles in the gastrointestinal system. The account that follows is offered as a biological background rather than as evidence of efficacy, as none of the included studies measured these mechanisms. The osmotic properties of poorly absorbed magnesium salts improve stool consistency by drawing water into the intestinal lumen, thereby increasing intraluminal volume and stimulating peristalsis [6,11]. Moreover, the physiological role of magnesium in the Mg-ATP complex provides energy for myosin cross-bridge cycling in smooth muscle [2,4], offering a mechanistic basis for improved colonic motility and increased stool frequency.

Moreover, magnesium plays a role in the upregulation of aquaporin-3 expression, which offers a candidate explanation for the improvements in consistency observed across studies. Aquaporin-3 is expressed in the basolateral membrane of colonic epithelial cells and mediates transepithelial water transport [25]. Its dysregulation may contribute to excessive colonic water absorption and the formation of hard stools. Ikarashi et al. [6] demonstrated that increasing intracellular Mg^2+^ concentration upregulates aquaporin-3 expression in colonic epithelial cells [6], and decreased aquaporin-3 expression has been independently linked to inflammatory gastrointestinal conditions, including IBS and IBD [7,8]. The evidence for this pathway derives from cellular and animal models, and aquaporin-3 expression was not measured in any of the included studies. It therefore remains a hypothesis rather than a demonstrated explanation for the shift from Bristol types 1-2 to types 3-4 observed in these trials. Furthermore, magnesium’s role in calcium homeostasis may influence both low-grade inflammatory processes increasingly attributed to functional gastrointestinal disorders and smooth muscle contractile function [5,26,27]. These pathways were not assessed in the included trials, and no causal link to clinical outcomes reported here can be inferred.

Clinical Implications

The clinical implications differ across population subgroups. In infants, the use of magnesium-enriched formulas was associated with rapid improvements in both stool frequency and consistency [16,21], although these data do not support a first-line recommendation. However, it must be noted that both formulas differed from controls in components other than magnesium, including whey hydrolysis, galacto-oligosaccharide content, and fat composition, precluding isolation of magnesium’s specific contribution. This suggests a promising area for further studies. In pediatric patients, Worona-Dibner et al. [18] demonstrated equivalent long-term efficacy between magnesium hydroxide and PEG 3350 (p = 0.616), consistent with magnesium hydroxide performing similarly to the guideline-recommended first-line osmotic laxative, although this is based on a single open-label trial [28,29].

In adults, the double-blind RCTs favored magnesium oxide at around 1,500 mg/day, with Morishita et al. [19] reporting 68.3% overall improvement responders versus 11.7% with placebo (p < 0.0001). These effect sizes are in a similar range to those reported for established pharmacological agents in chronic constipation, though this is based on indirect comparison [29]. It must be noted, however, that Kessoku et al. [20] showed magnesium oxide was inferior to elobixibat for CSBMs in a treatment-refractory population (p = 0.013), suggesting that while magnesium may be effective as a first-line osmotic agent, patients with refractory symptoms may require alternative treatment approaches. Another important consideration for clinical practice is the distinction between elemental magnesium and compound weight. For magnesium oxide, elemental magnesium accounts for 60% of the compound’s weight, a fact explicitly reported in only two studies [17,19], complicating dose comparisons and clinical dosing recommendations across studies. These observations describe the current evidence rather than supporting a treatment recommendation.

These findings should not be treated as evidence that all magnesium preparations have a uniform class effect. The studies used different magnesium compounds in different delivery vehicles, and none of the studies compared one magnesium preparation against another. Therefore, the efficacy of magnesium oxide cannot be assumed to transfer to magnesium hydroxide or other magnesium formulas. As only two studies explicitly reported elemental magnesium content, doses reported as compounds cannot be reliably converted to a common scale for comparison. Apparent differences in effect between formulations might reflect differences in the amount of elemental magnesium delivered. Future trials should report both the elemental magnesium dose and the compound weight, and direct head-to-head comparisons between different magnesium preparations remain a gap in the evidence.

Limitations

Several limitations must be considered for this study. There was substantial clinical heterogeneity across populations, formulations, dosing regimens, and treatment durations, which limits the generalizability of population-specific dose-response conclusions. Moreover, the included studies generally had small sample sizes, with only Benninga et al. [16] exceeding 100 participants. Quality assessment also revealed methodological concerns, including the absence of blinding in four of seven studies, baseline imbalances in key prognostic variables [15], and the absence of between-group comparisons in Kubota et al. [17]. Outcome measurement was generally inconsistent, with four different stool consistency instruments and validated quality-of-life assessments in only two studies [15,19]; future studies should adopt standardized instruments, such as the PAC-QOL [23]. Several interventions in the study were also confounded, including multi-component infant formulas [16,21] and the absence of a pure placebo arm [17]. Moreover, the studies were geographically concentrated in Japan, with four of the seven conducted in Japan. There was also a predominantly female adult sample (100% in Mori et al. [15]; 93.3% in Morishita et al. [19]), which limits the generalizability of the study’s findings. Limitations in the review process must be acknowledged, as the study was not prospectively registered, and the certainty of the evidence base was not formally graded utilizing a structured framework such as GRADE. Reporting and publication biases were not formally assessed, as the small number of included studies precluded funnel-plot or statistical evaluation. No quantitative synthesis was performed, and sensitivity analyses were not feasible. Requiring population, intervention, and outcome terms simultaneously may have reduced search sensitivity, and the search was restricted to a 10-year window and the five listed databases, without searches of trial registries, grey literature, or reference lists. Finally, as all the studies addressed functional constipation, the findings cannot be extrapolated to IBS-C or other FBDs.

## Conclusions

This systematic review provides preliminary evidence that magnesium supplementation, primarily as magnesium oxide, is associated with improvements in stool frequency, stool consistency, and constipation-specific quality of life in patients with functional constipation across infant, pediatric, and adult populations. The evidence base is limited by small sample sizes, varied study designs, inconsistent outcome measures, and confounded interventions, and its certainty was not formally graded. The findings relate to magnesium oxide and cannot be assumed to extend to other forms of magnesium supplementation. These findings should be considered preliminary and hypothesis-generating, with the aim of guiding further research in this domain. Future research should prioritize adequately powered, double-blind, placebo-controlled RCTs that clearly specify elemental versus compound dosing, employ standardized, validated outcomes, include treatment durations of at least 12 weeks with post-cessation follow-up, and incorporate mechanistic sub-studies examining colonic transit, aquaporin-3 expression, and serum magnesium levels. Diverse geographic and demographic recruitment is essential to establish generalizability.

## Appendices

**Table 4:** Search strategy for databases. All searches were run on December 16, 2025. Retrieval was restricted to primary human studies published between 2015 and 2025; date and document-type limits were applied using the native interface of each platform, so the operational form of these limits differs between databases. Records were exported to Covidence, where duplicates were removed and screening was managed.

Database	Platform
Scopus	Elsevier Scopus
PubMed	NCBI PubMed
Embase	Elsevier Embase
Web of Science	Clarivate Web of Science Core Collection
CINAHL	EBSCOhost

Set	Search query as entered
#1	(((((constipation) OR (irritable bowel syndrome)) OR (functional constipation)) OR (IBS-C)) OR (functional bowel disorders)) OR (functional bowel disorder)
#2	(((((magnesium) OR (magnesium oxide)) OR (magnesium citrate)) OR (magnesium hydroxide)) OR (magnesium sulfate)) OR (milk of magnesia)
#3	((((((((bowel movement) OR (bowel movements)) OR (bristol stool)) OR (stool form)) OR (stool frequency)) OR (stool consistency)) OR (straining)) OR (abdominal pain)) OR (quality of life)
#4	#1 AND #2 AND #3
#5	(Review[Title]) OR (guideline[Title])
#6	#4 NOT #5

Set	Search query as entered
#1	(((((TS=(constipation)) OR TS=(Irritable bowel syndrome)) OR TS=(functional constipation)) OR TS=(IBS-C)) OR TS=(functional bowel disorders)) OR TS=(functional bowel disorder)
#2	(((((TS=(magnesium)) OR TS=(magnesium oxide)) OR TS=(magnesium citrate)) OR TS=(magnesium hydroxide)) OR TS=(magnesium sulfate)) OR TS=(milk of magnesia)
#3	((((((((TS=(bowel movement)) OR TS=(bowel movements)) OR TS=(bristol stool)) OR TS=(stool form)) OR TS=(stool frequency)) OR TS=(stool consistency)) OR TS=(straining)) OR TS=(abdominal pain)) OR TS=(quality of life)
#4	#1 AND #2 AND #3

Set	Search query as entered
#1	((TITLE-ABS-KEY(constipation) OR TITLE-ABS-KEY(Irritable bowel syndrome) OR TITLE-ABS-KEY(functional constipation) OR TITLE-ABS-KEY(IBS-C) OR TITLE-ABS-KEY(functional bowel disorders) OR TITLE-ABS-KEY(functional bowel disorder)))
#2	((TITLE-ABS-KEY(magnesium) OR TITLE-ABS-KEY(magnesium oxide) OR TITLE-ABS-KEY(magnesium citrate) OR TITLE-ABS-KEY(magnesium hydroxide) OR TITLE-ABS-KEY(magnesium sulfate) OR TITLE-ABS-KEY(milk of magnesia)))
#3	((TITLE-ABS-KEY(bowel movement) OR TITLE-ABS-KEY(bowel movements) OR TITLE-ABS-KEY(bristol stool) OR TITLE-ABS-KEY(stool form) OR TITLE-ABS-KEY(stool frequency) OR TITLE-ABS-KEY(stool consistency) OR TITLE-ABS-KEY(straining) OR TITLE-ABS-KEY(abdominal pain) OR TITLE-ABS-KEY(quality of life)))

Set	Search query as entered
#1	(bowel movement OR bowel movements OR bristol stool OR stool form OR stool frequency OR stool consistency OR straining OR abdominal pain OR quality of life) AND (magnesium OR magnesium oxide OR magnesium citrate OR magnesium hydroxide OR magnesium sulfate OR milk of magnesia) AND (constipation OR Irritable bowel syndrome OR functional constipation OR IBS-C OR functional bowel disorders OR functional bowel disorder)

Set	Search query as entered
#1	Population: constipation OR irritable bowel syndrome OR functional constipation OR IBS-C OR functional bowel disorders OR functional bowel disorder
#2	Intervention: magnesium OR magnesium oxide OR magnesium citrate OR magnesium hydroxide OR magnesium sulfate OR milk of magnesia
#3	Outcomes: bowel movement OR bowel movements OR bristol stool OR stool form OR stool frequency OR stool consistency OR straining OR abdominal pain OR quality of life
#4	#1 AND #2 AND #3

**Table 5:** Full-text records were excluded at the eligibility stage, with reasons. Records [30-38] were retrieved as full text and checked against the eligibility criteria, then excluded for the reason shown. Where a study failed on more than one count, only the main reason is given. Studies dropped earlier, at title and abstract screening, are not listed here.

Study	Reason for exclusion
Comparing Golqand (Persian medicine product) with magnesium hydroxide in adult constipation: a randomised clinical trial [30]	Wrong comparator: magnesium hydroxide was compared with Golqand, a Persian medicine product, rather than with an agent in routine clinical use
Efficacy and safety of a natural mineral water rich in magnesium and sulphate for bowel function: a double-blind, randomised, placebo-controlled study. [31]	Wrong intervention: the product tested was a magnesium and sulphate-rich natural mineral water rather than a magnesium supplement
Health properties of the Italian San Martino® mineral-rich water: a self-controlled pilot study [32]	Wrong intervention: the study evaluated a mineral-rich drinking water (San Martino) rather than a defined magnesium supplement
Time to treatment response of a magnesium- and sulphate-rich natural mineral water in functional constipation. [33]	Wrong intervention: the exposure was a magnesium- and sulphate-rich mineral water rather than a magnesium supplement
Efficacy of oral magnesium therapy in the treatment of chronic constipation in spastic cerebral palsy children: a randomised controlled trial [34]	Wrong patient population: participants were children with spastic cerebral palsy, whose constipation is neurogenic rather than a functional bowel disorder
Effects of sulfate-rich mineral water on functional constipation: a double-blind, randomised, placebo-controlled study [35]	Wrong intervention: the intervention was a sulphate-rich mineral water rather than a magnesium supplement
Relationship between stool form and quality of life in patients with chronic constipation: an internet questionnaire survey [36]	Wrong study design: the study was a cross-sectional internet questionnaire survey, not a comparative interventional study
Severe hypermagnesemia induced by magnesium oxide ingestion: a case series [37]	Wrong study design: the report was a case series of hypermagnesemia rather than a comparative study
Factors associated with defecation satisfaction among Japanese adults with chronic constipation [38]	Wrong outcomes: the study examined factors associated with defecation satisfaction but did not report the prespecified stool or quality-of-life outcomes

**Table 6:** Preferred Reporting Items for Systematic Reviews and Meta-Analyses (PRISMA) 2020 checklist. Page et al. [13]. This work is licensed under CC BY 4.0. To view a copy of this license, visit https://creativecommons.org/licenses/by/4.0/.

Section and topic	Item #	Checklist item	Location where item is reported
Title			
Title	1	Identify the report as a systematic review.	Title page
Abstract			
Abstract	2	See the PRISMA 2020 for Abstracts checklist.	Abstract (Background, Methods, Results, Conclusions)
Introduction			
Rationale	3	Describe the rationale for the review in the context of existing knowledge.	Introduction, paragraphs 1-4
Objectives	4	Provide an explicit statement of the objective(s) or question(s) the review addresses.	Introduction, final paragraph
Methodology			
Eligibility criteria	5	Specify the inclusion and exclusion criteria for the review and how studies were grouped for the syntheses.	Methods, “Eligibility Criteria”
Information sources	6	Specify all databases, registers, websites, organisations, reference lists and other sources searched or consulted to identify studies. Specify the date when each source was last searched or consulted.	Methods, "Search Strategy" (five databases; 16 December 2015 to 16 December 2025; registries, grey literature and reference lists not searched; all searches run 16 December 2025; Supplementary Appendix Table 4)
Search strategy	7	Present the full search strategies for all databases, registers and websites, including any filters and limits used.	Supplementary Appendix Table 4 (full strategies as entered for PubMed, Web of Science, Scopus and CINAHL; Embase strategy reconstructed from the concept blocks)
Selection process	8	Specify the methods used to decide whether a study met the inclusion criteria of the review, including how many reviewers screened each record and each report retrieved, whether they worked independently, and if applicable, details of automation tools used in the process.	Methods, “Study Selection and Data Extraction”
Data collection process	9	Specify the methods used to collect data from reports, including how many reviewers collected data from each report, whether they worked independently, any processes for obtaining or confirming data from study investigators, and if applicable, details of automation tools used in the process.	Methods, “Study Selection and Data Extraction”
Data items	10a	List and define all outcomes for which data were sought. Specify whether all results that were compatible with each outcome domain in each study were sought (e.g. for all measures, time points, analyses), and if not, the methods used to decide which results to collect.	Methods, “Eligibility Criteria” and “Study Selection and Data Extraction”
	10b	List and define all other variables for which data were sought (e.g. participant and intervention characteristics, funding sources). Describe any assumptions made about any missing or unclear information.	Methods, “Study Selection and Data Extraction” (variables extracted listed, study authors not contacted, no imputations)
Study risk of bias assessment	11	Specify the methods used to assess risk of bias in the included studies, including details of the tool(s) used, how many reviewers assessed each study and whether they worked independently, and if applicable, details of automation tools used in the process.	Methods, “Quality Appraisal”
Effect measures	12	Specify for each outcome the effect measure(s) (e.g. risk ratio, mean difference) used in the synthesis or presentation of results.	Methods, “Data Synthesis” (no common effect measure calculated; study-reported estimates and P values presented as published)
Synthesis methods	13a	Describe the processes used to decide which studies were eligible for each synthesis (e.g. tabulating the study intervention characteristics and comparing against the planned groups for each synthesis (item #5)).	Methods, “Data Synthesis”; Table 1
	13b	Describe any methods required to prepare the data for presentation or synthesis, such as handling of missing summary statistics, or data conversions.	Methods, “Data Synthesis” (no data conversions or imputations performed)
	13c	Describe any methods used to tabulate or visually display results of individual studies and syntheses.	Methods, “Data Synthesis”; Tables 1-3
	13d	Describe any methods used to synthesize results and provide a rationale for the choice(s). If meta-analysis was performed, describe the model(s), method(s) to identify the presence and extent of statistical heterogeneity, and software package(s) used.	Methods, “Data Synthesis” (narrative synthesis; meta-analysis deemed inappropriate due to heterogeneity)
	13e	Describe any methods used to explore possible causes of heterogeneity among study results (e.g. subgroup analysis, meta-regression).	Methods, “Data Synthesis” (data organised by primary outcome, stratified by population subgroup)
	13f	Describe any sensitivity analyses conducted to assess robustness of the synthesized results.	Methods, “Data Synthesis” (sensitivity analyses considered but not feasible given the small number of heterogeneous studies)
Reporting bias assessment	14	Describe any methods used to assess risk of bias due to missing results in a synthesis (arising from reporting biases).	Methods, “Data Synthesis” (risk of bias due to missing results not formally assessed)
Certainty assessment	15	Describe any methods used to assess certainty (or confidence) in the body of evidence for an outcome.	Methods, “Data Synthesis”; Abstract; Limitations (certainty not formally graded; GRADE not applied)
Results			
Study selection	16a	Describe the results of the search and selection process, from the number of records identified in the search to the number of studies included in the review, ideally using a flow diagram.	Results, “Search Results and Study Characteristics”, paragraph 1; Figure 1
	16b	Cite studies that might appear to meet the inclusion criteria, but which were excluded, and explain why they were excluded.	Results, “Search Results and Study Characteristics”, paragraph 1 (exclusions by reason); Supplementary Appendix Table 6 (excluded records cited with reasons)
Study characteristics	17	Cite each included study and present its characteristics.	Results, “Search Results and Study Characteristics”, paragraph 1; Table 1
Risk of bias in studies	18	Present assessments of risk of bias for each included study.	Results, “Quality Assessment”; Table 2
Results of individual studies	19	For all outcomes, present, for each study: (a) summary statistics for each group (where appropriate) and (b) an effect estimate and its precision (e.g. confidence/credible interval), ideally using structured tables or plots.	Results, “Stool Frequency,” “Stool Consistency,” “Quality of Life,” “Adverse Events”; Table 3
Results of syntheses	20a	For each synthesis, briefly summarise the characteristics and risk of bias among contributing studies.	Results, “Quality Assessment”; Discussion, paragraph 2; Tables 1-2
	20b	Present results of all statistical syntheses conducted. If meta-analysis was done, present for each the summary estimate and its precision (e.g. confidence/credible interval) and measures of statistical heterogeneity. If comparing groups, describe the direction of the effect.	Results, outcome subsections; Discussion, “Summary of Evidence” (narrative synthesis; no statistical synthesis performed)
	20c	Present results of all investigations of possible causes of heterogeneity among study results.	Discussion, paragraph 2
	20d	Present results of all sensitivity analyses conducted to assess the robustness of the synthesized results.	Methods, “Data Synthesis” (sensitivity analyses considered but not feasible; none conducted)
Reporting biases	21	Present assessments of risk of bias due to missing results (arising from reporting biases) for each synthesis assessed.	Methods, “Data Synthesis”; Limitations (not formally assessed; small number of included studies precluded funnel-plot or statistical evaluation)
Certainty of evidence	22	Present assessments of certainty (or confidence) in the body of evidence for each outcome assessed.	Abstract; Limitations; Conclusions (not formally assessed)
Discussion			
Discussion	23a	Provide a general interpretation of the results in the context of other evidence.	Discussion, “Summary of Evidence,” “Mechanism of Action,” “Clinical Implications”
	23b	Discuss any limitations of the evidence included in the review.	Limitations
	23c	Discuss any limitations of the review processes used.	Limitations
	23d	Discuss implications of the results for practice, policy, and future research.	Discussion, “Clinical Implications”; Conclusions, final paragraph
Other information			
Registration and protocol	24a	Provide registration information for the review, including register name and registration number, or state that the review was not registered.	Methods, “Protocol and Registration” (not registered with PROSPERO)
	24b	Indicate where the review protocol can be accessed, or state that a protocol was not prepared.	Methods, “Protocol and Registration” (protocol finalised December 2025; held institutionally, not publicly accessible)
	24c	Describe and explain any amendments to information provided at registration or in the protocol.	Methods, “Protocol and Registration” (protocol finalised December 2025 without amendment)
Support	25	Describe sources of financial or non-financial support for the review, and the role of the funders or sponsors in the review.	Front matter, Funding (no funding received)
Competing interests	26	Declare any competing interests of review authors.	Front matter, Conflicts of Interest (none declared)
Availability of data, code and other materials	27	Report which of the following are publicly available and where they can be found: template data collection forms; data extracted from included studies; data used for all analyses; analytic code; any other materials used in the review.	Methods, “Study Selection and Data Extraction” (data derived from the published studies cited; no template data-collection form or analytic code generated)
Abstract checklist			
Title			
Title	1	Identify the report as a systematic review.	Yes (title: “A Systematic Review”)
Background			
Objectives	2	Provide an explicit statement of the main objective(s) or question(s) the review addresses.	Yes (Abstract, paragraph 1, final sentence)
Methodology			
Eligibility criteria	3	Specify the inclusion and exclusion criteria for the review.	Yes (Abstract, paragraph 1, final sentence: population, intervention, comparators and outcomes)
Information sources	4	Specify the information sources (e.g. databases, registers) used to identify studies and the date when each was last searched.	Yes (Abstract, paragraph 2: five databases; 10-year period to December 2025)
Risk of bias	5	Specify the methods used to assess risk of bias in the included studies.	Yes (Abstract, paragraph 2: MMAT)
Synthesis of results	6	Specify the methods used to present and synthesise results.	Yes (Abstract, paragraph 2: narrative synthesis due to heterogeneity)
Results			
Included studies	7	Give the total number of included studies and participants and summarise relevant characteristics of studies.	Yes (Abstract, paragraph 3: seven studies, n = 690; populations and study designs summarised)
Synthesis of results	8	Present results for main outcomes, preferably indicating the number of included studies and participants for each. If meta-analysis was done, report the summary estimate and confidence/credible interval. If comparing groups, indicate the direction of the effect (i.e. which group is favoured).	Yes (Abstract, paragraphs 3-4: study counts and direction of effect per outcome; no meta-analysis conducted)
Discussion			
Limitations of evidence	9	Provide a brief summary of the limitations of the evidence included in the review (e.g. study risk of bias, inconsistency and imprecision).	Yes (Abstract, paragraphs 2 and 5: certainty not formally graded; small samples, heterogeneous designs, confounded interventions)
Interpretation	10	Provide a general interpretation of the results and important implications.	Yes (Abstract, paragraph 5)
Other			
Funding	11	Specify the primary source of funding for the review.	No
Registration	12	Provide the register name and registration number.	No
